# Synthesis and Chemometrics of Thymol and Carvacrol Derivatives as Larvicides against *Aedes aegypti*

**Published:** 2017-05-27

**Authors:** Viviane Barros Silva, Daniele Lima Travassos, Angelita Nepel, Andersson Barison, Emmanoel Vilaça Costa, Luciana Scotti, Marcus Tulius Scotti, Francisco Jaime Bezerra Mendonça-Junior, Roseli La Corte dos Santos, Sócrates Cabral de Holanda Cavalcanti

**Affiliations:** 1 Medicinal Chemistry Laboratory, Pharmacy Department, Federal University of Sergipe, São Cristóvão, Brazil; 2 Nuclear Magnetic Resonance Laboratory, Chemistry Department, Federal University of Paraná, Curitiba, Brazil; 3 Chemistry Department, Institute of Sciences, Federal University of Amazonas, Manaus, Brazil; 4 Biotechnology Center, Federal University of Paraíba, Campus I, João Pessoa, Brazil; 5 Laboratory of Synthesis and Delivery of Molecules, Biological Sciences Department, State University of Paraíba, João Pessoa, Paraiba, Brazil; 6 Parasitology and Tropical Entomology Laboratory, Morphology Department, Federal University of Sergipe, São Cristóvão, Brazil

**Keywords:** Chemometry, SAR, Dengue, Thymol derivatives, Carvacrol derivatives

## Abstract

**Background:**

Thymol and carvacrol have previously demonstrated larvicidal activity against *Aedes aegypti* (Diptera: Culicidae). In view of this fact, it was of our interest to obtain synthetic derivatives and evaluate their larvicidal activity on *Ae. aegypti* larvae.

**Methods:**

Structural modifications were performed on thymol and carvacrol in an effort to understand the functional groups necessary for modulating their activities and to lead possibly to more effective larvae control agents. The derivatives were further subjected to SAR and computational studies (molecular modeling and chemometric tools (CPCA and PCA)) to extract structural information regarding their larvicidal properties. Field collected and Rockefeller populations of *Ae. aegypti* were used.

**Results:**

Carvacrol and thymol exhibited LC_50_ of 51 and 58ppm for field collected larvae, respectively. Carvacrol derivatives exhibited LC_50_ ranging from 39 to 169ppm, while thymol derivatives exhibited LC_50_ ranging from 18 to 465ppm. Substitution of the acidic proton of carvacrol by esters, ethers, and acetic acid resulted in either maintenance or reduction of potency.

**Conclusion:**

Thymol derivatives were, to a certain extent, more efficient larvicides against *Ae. aegypti* than carvacrol derivatives, particularly to Rockefeller larvae. The chemometrics tools applied in this study showed that the independent variables indicate a mixed profile. Nevertheless, hydrophobic interactions increased the larvicidal activity.

## Introduction

*Aedes aegypti* Linnaeus (Diptera: Culicidae), vector of dengue fever, is considered the most important disease-carrying mosquito in the world and has become a major international public health concern (WHO 2015). Unlike most vectors, *Ae. aegypti* lives near human habitation and breeds in a variety of water containers. During its life cycle, the female mosquitoes need blood as a source for supplemental substances, such as protein and iron to drive oogenesis (Gonçalves 2009). Contaminated mosquitoes transfer dengue virus from its salivary glands to humans during the course of the bite. After infection, the virus incubates for about six days resulting in a flu-like illness that affects infants, young children and adults, rarely causing death (Nishiura 2007). However, severe dengue is a complication characterized by high fever, which may progress to circulatory failure and death.

Because there is no treatment or vaccine available for treating or controlling dengue fever, the only available method to control dengue fever is to eliminate its vector, either by environmental actions, such as elimination of its breeding sites or by the use of pesticides. The use of larvicides is the most successful method to control mosquito infestation. Three major classes of larvicides are approved for use in potable water, organophosphates (e.g. temephos), growth regulators (eg juvenile hormone mimics and chitin synthesis inhibitors), and bacteria toxins (eg *Bacillus thuringiensis*, Bti) (WHO 2006). However, resistance to larvicides has stimulated researchers to develop new substances with different mechanism of action to control *Ae. aegypti* spreading (Polson 2011, Agra-Neto 2014).

A potential source for new pesticide candidates are natural product derivatives. Botanical chemical derivatives may not only be selective to a target species, but also more environmentally friendly than synthetic compounds. Monoterpenes have been chemically modified and evaluated against adult *Ae. aegypti* mosquitoes (Samarasekera 2008), as well as larvae (Santos 2010). Such studies have demonstrated the importance of double bonds and phenolic hydroxyls in the larvicidal potency (Santos 2010, Santos 2011, Lima 2013).

Thymol and carvacrol are important phenolic monoterpenoids obtained from the essential oil of plants. Both monoterpenes exhibit a large number of pharmacological activities, such as antimicrobial, antitumor, antiplatelet, analgesic, anti-inflammatory, antiangiogenic, and insecticidal (Baser 2008). Additionally, thymol and carvacrol are Generally Regarded As Safe (GRAS) food flavoring, which is an indication of low mammalian toxicity starting materials (US-OFR 2009).

The larvicidal efficiency of both thymol and carvacrol (Santos 2010) promoted us to undertake the synthesis of natural monoterpenoids derivatives. Most structural modifications were performed in the monoterpenoids phenolic moiety, resulting in a diverse set of groups with polar (acetic acid derivatives) and bulky/hydrophobic (ethers and esters) characteristics. Additionally, a relatively reactive aldehyde group was placed in the phenolic hydroxyl ortho position with the goal to verify the contribution of a relatively unstable moiety to the larvicidal activity. Furthermore, the larvicidal activities of thymol, carvacrol, and their derivatives against *Ae. aegypti* were assessed.

We have previously used Principal Component Analysis (PCA), Partial Least Squares Regression (PLS), and Consensus PCA (CPCA), to investigate a set of fifty-five compounds with activity against *Ae. aegypti* larvae. The PCA scores plot exhibited reasonable division between more and less potent compounds. The independent variables affected by a hydrophobic profile were sturdily correlated to the biological activity (Scotti 2014). To corroborate such findings the derivatives were further subjected to molecular modeling, chemometrics tools (PCA and CPCA), and SAR studies in order to extract structural information relating to their larvicidal properties.

## Materials and Methods

### General

Melting points were determined on a Logen Scientific melting point apparatus and are uncorrected. NMR data were recorded in CDCl_3_ or CD_3_OD at 293 K on a Bruker AVANCE 400 NMR spectrometer operating at 9.4 Tesla, observing ^1^H and ^13^C at 400 and 100MHz, respectively. All ^1^H and ^13^C NMR chemical shifts (δ) are given in ppm related to the TMS signal at 0.00ppm as internal reference. Coupling constants (J) are reported in hertz (Hz). The abbreviations used are s (singlet), d (doublet), t (triplet), q (quadruplet), m (multiplet), and sept (septuplet). FT-IR spectra were acquired on a Bomem MB-100 spectrophotometer. Mass spectra were recorded either on a Shimadzu GCMS-QP2010S Gas Chromatograph Mass Spectrometer (equipped with an AOC-20S autosampler) operating in positive mode or on an ion trap LCQ Fleet TM (Thermo Scientific) equipped with an EI source operating in negative mode. HRMS were recorded on a Bruker micrOTOF II-ESI-TOF mass spectrometer operating in positive mode. Reactions were monitored by thin-layer chromatography (TLC) using suitable mobile phase eluents, and visualization of the TLC plate was accomplished with UV light 256nm. Details of the physicochemical properties of the synthesized compounds are described below. General chemicals were obtained from Sigma-Aldrich Chemical Co. (St Louis, MO, USA).

**Fig. 4.**
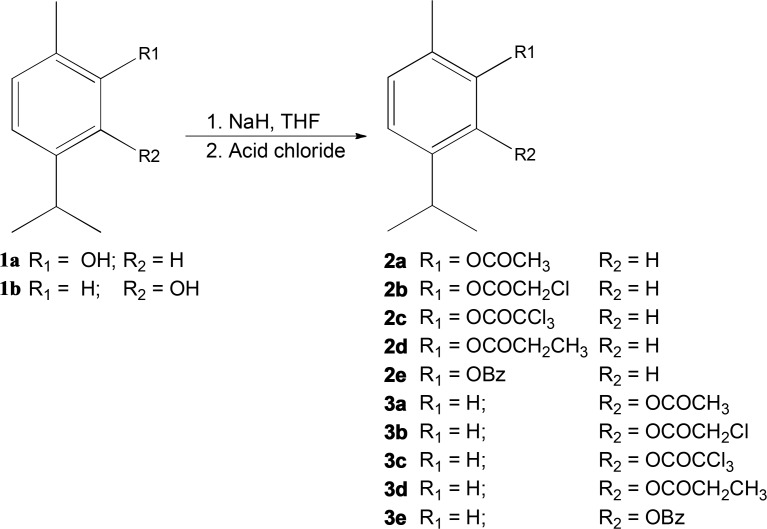
Synthesis of carvacrol and thymol esters

**Fig. 6.**
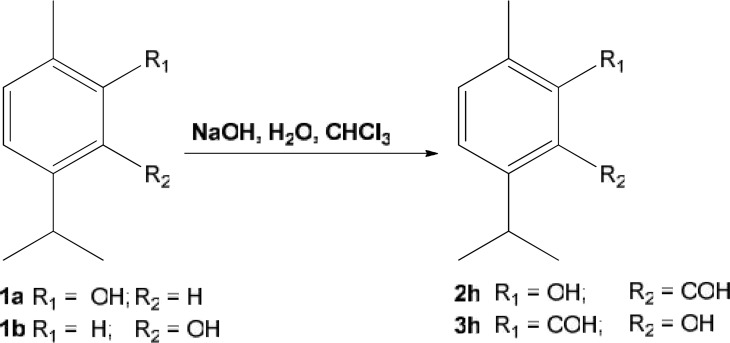
Synthesis of 2-hydroxy-3-methyl-6-(1-methylethyl)-benzaldehyde and 2-hydroxy-6-methyl-3-(1-methylethyl)-benzaldehyde

**Fig. 7.**
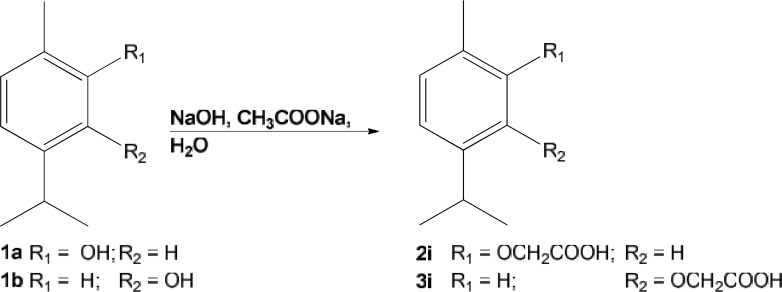
Synthesis of carvacrylglycolic and thymoxyacetic acids

Carvacryl benzoate was synthesized (Nikumbh 2003) using BzCl in the presence of aqueous 10% NaOH at room temperature (Fig. 4). Ether reactions were carried out using parent phenols (50mmol) and the corresponding alkyl iodide (60mmol) in the presence of K_2_CO_3_ (120mmol) using acetone as solvent, under reflux (Coolen 1995). 2-Hydroxy-3-methyl-6-(1-methylethyl)-benzaldehyde and 2-hydroxy-6-methyl-3-(1-methylethyl)-benzaldehyde were synthesized according to the procedure of Singh et al. (Singh 1989) by a Reimer-Tiemann reaction (Fig. 6). Carvacrylglycolic and thymoxyacetic acids were synthesized using parent phenols, chloroacetic acid, and NaOH dissolved in water, heated to reflux overnight (Fig. 7) (Varma 1985, Riedl 1998, Nikumbh 2003).

### General method for the synthesis of carvacryl and thymyl esters

Parent phenols (50mmol) were added to the corresponding anhydride or acid chloride (60mmol) to form ester derivatives in the presence of base (sodium acetate, triethylamine or NaH, 60mmol). THF was used as the solvent, and the reaction was allowed to stir for 1–4h at room temperature. The reaction mixture was concentrated under vacuum, diluted with water and extracted with dichloromethane. The organic layer was washed with water, and dried over Na_2_SO_4_. The solvent was distilled off and the residue purified by silica gel column chromatography (0.5% EtOAc: hexanes) (Dolly 2003, Ben Arfa 2006, Morais 2014).

### General method for the synthesis of carvacryl and thymyl ethers

**Fig. 5.**
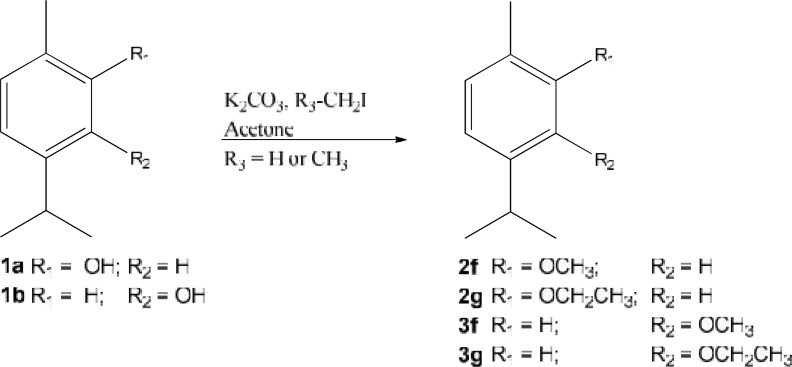
Synthesis of carvacrol and thymol ethers

Parent phenols (50mmol) and the corresponding alkyl iodide (60mmol) in the presence of K_2_CO_3_ (120mmol) and acetone as solvent, were stirred under reflux for 24h. The solvent was removed under reduced pressure, and the product was extracted in a CH_2_C1_2_/water mixture. The combined organic layers were washed three times with 1 N aqueous NaOH. The organic layer was separated, dried (MgSO_4_), and evaporated to dryness. The resulting oil was purified by flash chromatography over a silica gel column with hexanes as eluent to yield clear oils (Fig. 5) (Coolen 1995).

### General method for the synthesis of 2-Hydroxy-3-methyl-6-(1-methylethyl) benzaldehyde and 2-hydroxy-6-methyl-3-(1-methylethyl) benzaldehyde

A mixture of Carvacrol or thymol (50 mmol), NaOH (250mmol) and water (20mL) was heated to 60–70 °C. A refluxing condenser was connected to the reaction flask and chloroform (124mmol) was added dropwise. Extraction with EtOAc followed by drying (MgSO_4_) and evaporation of the solvent yielded yellowish oils, which were purified by silica gel column chromatography (hexanes) to afford the corresponding aldehydes (Knight 2005).

### General method for the synthesis of carvacrylglycolic and thymoxyacetic acids

Parent phenols (30mmol), chloroacetic acid (30mmol), and NaOH (61mmol) dissolved in 15mL of water were heated to reflux overnight. The solution was allowed to cool to room temperature, acidified to pH 1 with concentrated HCl with constant and vigorous stirring, and the precipitate was separated by filtration (Riedl 1998).

### Rearing of *Ae. aegypti*

Eggs of *Ae. aegypti* were field collected in Aracaju City, Sergipe state, Brazil and laboratory-reared at the Federal University of Sergipe insectary, at 27 °C and 80–85% relative humidity under a 12:12h light: dark cycle. *Aedes aegypti* collected in this neighborhood are resistant to temephos, a frequently used insecticide for larvae control. Eggs of Rockefeller were kindly donated by Laboratório de Fisiologia e Controle de Artrópodes Vetores (LAFICAVE, FIOCRUZ-Rio de Janeiro, Brazil). The *Ae. aegypti* Rockefeller strain is susceptible to temephos. Adults were provided with a 10% sucrose solution ad libitum. Assays eggs were obtained attached to paper strips. The paper strips (1000 eggs/L) were placed in a rectangular polyethylene container with natural mineral water. The container was kept in the insectary for hatching and monitoring of larvae development for three to four days. Larvae were fed with cat food (PurinaTM) to allow regular development. All bioassays were conducted in a walk-in environmental chamber with the above environmental conditions.

### Larvicidal assay

The larvicidal assay was performed according to Santos et al. (Santos 2010). In summary, third-instar larvae were used in the experiment. The concentration ranges were determined by a previous curve concentration-response with 20 larvae. A 20,000ppm stock solution was prepared using each compound (20mg/mL), Tween-80 (10%v/v), DMSO (30% v/v), and natural mineral water (60%v/v). The stock solution was used to make 20mL water solutions with concentrations preset by the concentration-response curve. Twenty larvae were collected with a Pasteur pipette, placed on a 25mL graduated cylinder. The volume was completed to 20mL with natural mineral water and transferred to disposable cups containing variable volumes of the stock solution. A mortality count was conducted 24h after treatment. Controls were prepared with Tween-80 (0.1mL), DMSO (0.3mL), and water (19.6mL). Three replicates were used for each concentration and the control.

### Statistics

Probit analysis (Finney 1971) was conducted on mortality data collected after 24h exposure to different concentration of testing solutions to establish the lethal concentration for 50% mortality (LC_50_) and 95% confidence interval values for the respective compounds (Tables 1, 2). In all cases where deaths had occurred in the control experiment, the data was corrected using Abbott’s formula (% Deaths= [1-(test/control)] ×100). Compounds activity is considered significantly different when the 95% CI fails to overlap.

**Table 1.** Larvicidal activities (LC_50_) and 95% confidence intervals (CI) of carvacrol and its derivatives on third-instar larvae of *Ae. Aegypti*

**Compound name**	**LC_50_ ppm (CI) Field collected**	**LC_50_ ppm (CI) Rockefeller**
Carvacrol, 1a	51 (48 to 55)	47 (43 to 50)
Carvacryl acetate, 2a	93 (84 to 104)	72 (65 to 79)
Carvacryl chloroacetate, 2b	52 (44 to 59)	39 (37 to 42)
Carvacryl trichloroacetate, 2c	77 (68 to 86)	54 (47 to 60)
Carvacryl propionate, 2d	66 (57 to 77)	77 (70 to 83)
Carvacryl benzoate, 2e	58 (49 to 69)	57 (45 to 72)
Carvacryl methyl ether, 2f	136 (116 to 154)	60 (52 to 69)
Carvacryl ethyl ether, 2g	120 (100 to 148)	67 (57 to 76)
2-Hydroxy-3-methyl-6-(1-methylethyl)-benzaldehyde, 2h	66 (58 to 75)	56 (51 to 61)
Carvacrylglycolic acid, 2i	169 (158 to 180)	113 (101 to 123)

**Table 2.** Larvicidal activities (LC_50_) and 95% confidence intervals (CI) of thymol and its derivatives on third-instar larvae of *Ae. Aegypti*

**Compound name**	**LC_50_ ppm (IC) Field collected**	**LC_50_ ppm (IC) Rockefeller**
Thymol, 1b	58 (54 to 63)	46 (41 to 52)
Thymyl acetate, 3a	93 (82 to 103)	79 (69 to 89)
Thymyl chloroacetate, 3b	49 (46 to 53)	25 (22 to 28)
Thymyl trichloroacetate, 3c	41 (37 to 45)	45 (41 to 51)
Thymyl propionate, 3d	66 (61–73)	45 (39 to 51)
Thymyl benzoate, 3e	90 (82 to 99)	18 (14 to 25)
Thymyl methyl ether, 3f	192 (169 to 214)	39 (33 to 48)
Thymyl ethyl ether, 3g	123 (29 to 269)	58 (50 to 65)
2-hydroxy-6-methyl-3-(1-methylethyl)-benzaldehyde, 3h	34 (32 to 37)	36 (32 to 41)
Thymoxyacetic acid, 3i	465 (426–513)	101 (95 to 107)

### Computational methods

The results of field collected larvae activity were used on computational methods.

### Molecular Modeling

The chemical structures of the compounds were drawn using the program Hyperchem v. 8.0 (Hypercube 2009), and their geometry was optimized using MM+ force field (Allinger 1977). Subsequently, a new geometry optimization based on the semi-empirical method AM1 (Austin Model 1) was performed (Dewar 1993). The optimized structures were subjected to conformational analyses using the random search method (Allinger 1977, Cohen 1996, Leach 2001) with 1000 interactions, 100 cycles of optimization, and 10 conformers of lowest minimum energy. The selected dihedrals were evaluated by rotation in accordance with the standard (default) conditions of the program, in which the number of simultaneous variations was 1 to 8, acyclic chains were submitted to rotations from 60 to 180° and torsion rings were in the range of 30 to 120°.

### Chemometrics

Structures modeled as described above were used to calculate the molecular descriptors via VolSurf+ program. PCA and CPCA methodologies were applied to the set of derivatives using the VolSurf+ software (Cruciani 2000, Cruciani 2000, Cruciani 2002, Cianchetta 2004). The LC_50_ of the derivatives were converted to molar unitary and then expressed in negative logarithmic units, pLC_50_ (−log LC_50_).

### CPCA (Consensus PCA)

A preliminary exploratory analysis, CPCA, which considered 128 independent variables or descriptors, was developed. Pre-processing (autoscaling) of data was performed, and 13 blocks of descriptors were calculated.

### PCA

With regard to the interaction of 3D structures and a GRID force field, PCA results were obtained using the H2O, DRY and LogS probes. Fifty-four descriptors were selected from CPCA. The data were autoscaled (pre-processed).

## Results

### Synthesis

Twenty monoterpenes were synthesized, characterized, and evaluated. Spectroscopic data are depicted below.

### Carvacryl acetate (2a)

Was prepared from carvacrol using the method of Ben Arfa et al. (2006). Clear oil (75%). IR (film, cm^−1^) 2960 (C-H), 1766 (C=O), 1215 (-CC=OO-). ^1^H NMR (400 MHz, CDCl_3_) δ 7.11 (d, 1H, J= 7.8 Hz, Ar-H), 6.98 (d, 1H, J= 7.8 Hz, Ar-H), 6.85 (s, 1H, Ar-H), 2.85 (sept, 1H, J= 6.9 Hz, CH(CH_3_)_2_), 2.25 (s, 3H, COCH_3_), 2.11 (s, 3H, Ar-CH_3_), 1.21 (d, 6H, J= 6.9 Hz, CH(CH_3_)_2_). ^13^C NMR (100 MHz, CDCl_3_): δ 169.2, 149.3, 148.0, 130.8, 127.1, 124.1, 119.7, 33.5, 23.9, 20.7, 15.7. MS (EI) m/z [M]^+^ 192.

### Carvacryl chloroacetate (2b)

Was prepared from carvacrol using the method of Dolly et al. (2003). Yellowish oil (78%). IR (film, cm^−1^) 2960 (C-H), 1778 (C=O), 1238 (CH_2_Cl), 1145 (-CC=OO-), 705 (C-Cl). ^1^H NMR (400 MHz, CDCl_3_) δ 7.13 (d, 1H, J= 7.8 Hz, Ar-H), 7.02 (d, 1H, J= 7.8 Hz, Ar-H), 6.89 (s, 1H, Ar-H), 4.25 (s, 2H, CH_2_ Cl), 2.86 (sept, 1H, J= 6.9 Hz, CH(CH_3_)_2_), 2.13 (s, 3H, Ar-CH_3_), 1.21(d, 6H, J= 6.9 Hz, CH(CH_3_)_2_). ^13^C NMR (100 MHz, CDCl_3_): δ 165.6, 148.8, 148.2, 131.0, 126.9, 124.6, 119.3, 40.7, 33.5, 23.8, 15.6. HRMS calcd for C_12_H_15_ClO_2_Na (M+Na)^+^ m/z 249.0658, found 249.0659.

### Carvacryl trichloroacetate (2c)

Was prepared from carvacrol using the method of Dolly et al. (2003). Yellowish oil (84%). IR (film, cm^−1^) 2960 (C-H), 1776 (C=O), 1219 (-CC=OO-), 677 (C-Cl). ^1^H NMR (400 MHz, CDCl_3_) δ 7.16 (d, 1H, J= 7.8 Hz, Ar-H), 7.07 (d, 1H, J= 7.8 Hz, Ar-H), 6.97 (s, 1H, Ar-H), 2.88 (sept, 1H, J= 6.9 Hz, CH (CH_3_)_2_), 2.21 (s, 3H, Ar-CH_3_), 1.23 (d, 6H, J= 6.9 Hz, CH(CH_3_)_2_). ^13^C NMR (100 MHz, CDCl_3_): δ 160.2, 148.9, 148.5, 131.3, 126.7, 125.2, 118.5, 89.8, 33.6, 23.8, 15.4. HRMS calcd for C_12_H_13_Cl_3_O_2_Na (M+Na)^+^ m/z 316.9879, found 316.9875.

### Carvacryl propionate (2d)

Was prepared from carvacrol using the method of Dolly et al. (2003). Clear oil (72%). IR (film, cm cm^−1^) 2960 (C-H), 1760 (C=O), 1147 (-CC=OO-). ^1^H NMR (400 MHz, CDCl_3_) δ 7.10 (d, 1H, J= 7.8 Hz, Ar-H), 6.98 (d, 1H, J= 7.8 Hz, Ar-H), 6.85 (s, 1H, Ar-H), 2.84 (sept, 1H, J= 6.8 Hz, CH(CH_3_)_2_), 2.56 (q, 2H, J= 7.6 Hz, CO-CH_2_-CH_3_), 2.10 (s, 3H, Ar-CH_3_), 1.25 (t, 3H, J= 7.5 Hz, CO-CH_2_-CH_3_), 1.21 (d, 6H, J= 6.9 Hz, CH(CH_3_)_2_). ^13^C NMR (100 MHz, CDCl_3_): δ 172.5, 149.3, 147.9, 130.8, 127.1, 124.0, 119.7, 33.6, 27.6, 23.9, 15.7, 9.2. MS (EI) m/z [M]^+^ 206.

### Carvacryl benzoate (2e)

Was prepared from carvacrol using the Schotten-Baumann reaction. (Morais 2014). Clear oil (73%). IR (film, cm^−1^) 2960 (C-H), 1737 (C=O), 1263 (-CC=OO-). ^1^H NMR (400 MHz, CDCl_3_) δ 8.21 (d, 2H, J= 8.4 Hz, Ar-H), 7.56 (t, 1H, J= 7.4 Hz, Ar-H), 7.47-7.43 (m, 2H, Ar-H), 7.16 (d, 1H, J= 7.7 Hz, Ar-H), 7.02 (d, 1H, J= 7.7 Hz, Ar-H), 7.00 (s, 1H, Ar-H), 2.87 (sept, 1H, J= 6.9 Hz, CH(CH_3_)_2_), 2.17 (s, 3H, Ar -CH_3_), 1.23 (d, 6H, J= 6.9 Hz, CH(CH_3_)_2_). ^13^C NMR (100 MHz, CDCl_3_): δ 164.8, 149.5, 148.0, 133.4, 130.9, 130.1, 129.6, 128.5, 127.3, 124.1, 119.8, 33.6, 23.9, 15.8. MS (EI) m/z [M]^+^ 254.

### Thymyl acetate (3a)

Was prepared from thymol using the method of Ben Arfa et al. (2006). Clear oil (93%). IR (film, cm^−1^) 2966 (C-H), 1760 (C=O), 1220 (-CC=OO-). ^1^H NMR (400 MHz, CDCl_3_) δ 7.18 (d, 1H, J= 7.9 Hz, Ar-H), 7.01 (d, 1H, J= 7.9 Hz, Ar-H), 6.80 (s, 1H, Ar-H), 2.96 (sept, 1H, J = 6.9 Hz, CH(CH_3_)_2_), 2.30 (s, 6H, OCOCH_3_ and Ar-CH_3_), 1.18 (d, 6H, J= 6.9 Hz, CH(CH_3_)_2_). ^13^C NMR (100 MHz, CDCl_3_): δ 169.7, 147.8, 136.9, 136.5, 127.1, 126.4, 122, 7, 27.1, 23.0, 20.9, 20.8. MS (EI) m/z [M]^+^ 192.

### Thymyl chloroacetate (3b)

Was prepared from thymol using the method of Dolly et al. (2003). Yellowish oil (62%). IR (film, cm^−1^) 2960 (C-H), 1776 (C=O), 1240 (CH_2_Cl), 1157 (-CC=OO-), 779 (C-Cl). ^1^H NMR (400 MHz, CDCl_3_) δ 7.19 (d, 1H, J= 7.9 Hz, Ar-H), 7.02 (d, 1H, J= 7.9 Hz, Ar-H), 6.82 (s, 1H, Ar-H), 4.24 (s, 2H, CH_2_ Cl), 2.97 (sept, 1H, J= 6.9 Hz, CH(CH_3_)_2_), 2.28 (s, 3H, Ar-CH_3_), 1.18(d, 6H, J= 6.9 Hz, CH(CH_3_)_2_). ^13^C NMR (100 MHz, CDCl_3_): δ 166.1, 147.5, 136.8, 136.7, 127.6, 126.6, 122.2, 40.7, 27.0, 23.0, 20.7. MS (EI) m/z [M]^+^ 226.

### Thymyl trichloroacetate (3c)

Was prepared from thymol using the method of Dolly et al. (2003). Yellowish oil (90%). IR (film, cm^−1^) 2964 (C-H), 1776 (C=O), 1224 (-CC=OO-), 676 (C-Cl). ^1^H NMR (400 MHz, CDCl_3_) δ 7.16 (d, 1H, J= 7.8 Hz, Ar-H), 7.07 (d, 1H, J= 7.8 Hz, Ar-H), 6.97 (s, 1H, Ar-H), 2.88 (sept, 1H, J= 6.9 Hz, CH(CH_3_)_2_), 2.21 (s, 3H, Ar-CH_3_), 1.23 (d, 6H, J= 6.9 Hz, CH(CH_3_)_2_). ^13^C NMR (100 MHz, CDCl_3_): δ 160.2, 148.9, 148.5, 131.3, 126, 7, 125.2, 118.5, 89.8, 33.5, 23.8, 15.4. HRMS calcd for C_12_ H_13_Cl_3_O_2_Na (M+Na)^+^ m/z 316.9879, found 316.9870.

### Thymyl propionate (3d)

Was prepared from thymol using the method of Dolly et al. (2003). Clear oil (68%). IR (film, cm^−1^) 2966 (C-H), 1758 (C=O), 1149 (-CC=OO-). ^1^H NMR (400 MHz, CDCl_3_) δ 7.17 (d, 1H, J= 7.9 Hz, Ar-H), 6.98 (d, 1H, J= 7.9 Hz, Ar-H), 6.79 (s, 1H, Ar-H), 2.96 (sept, 1H, J= 6.8 Hz, CH(CH_3_)_2_), 2.57 (q, 2H, J= 7.5 Hz, COCH_2_CH_3_), 2.28 (s, 3H, Ar-CH_3_), 1.26 (t, 3H, J= 7.6 Hz, COCH_2_CH_3_), 1.18 (d, 6H, J= 6.9 Hz, CH(CH_3_)_2_). ^13^C NMR (100 MHz, CDCl_3_): δ 173.0, 148.0, 136.9, 136.4, 127.0, 126.3, 122.7, 27.7, 27.1, 23.0, 20.7, 9.2. MS (EI) m/z [M]^+^ 206.

### Thymyl benzoate (3e)

Was prepared from thymol using the Schotten-Baumann reaction. (Morais 2014). White crystals (94%), mp 28–29 °C. IR (KBr, cm^−1^) 2958 (C-H), 1735 (C=O), 1236 (-CC=OO-). ^1^H NMR (400 MHz, CDCl_3_) δ 8.22 (d, 2H, J= 7.1 Hz, Ar-H), 7.62 (t, 1H, J= 7.4 Hz, Ar-H), 7.50 (t, 2H, J= 7.3 Hz, Ar-H), 7.24 (d, 1H, J= 7.9 Hz, Ar-H), 7.06 (d, 1H, J= 7.9 Hz, Ar-H), 6.94 (s, 1H, Ar-H), 3.05 (sept, 1H, J= 6.8 Hz, CH(CH_3_)_2_), 2.33 (s, 3H, Ar-CH_3_), 1.21 (d, 6H, J= 6.9 Hz, CH(CH_3_)_2_). ^13^C NMR (100 MHz, CDCl_3_): δ 165.3, 148.1, 137.1, 136.6, 133.5, 130.1, 129.6, 128.6, 127.1, 126.4, 122.8, 27.2, 23.0, 20.8. MS (EI) m/z [M]^+^ 254.

### Carvacryl methyl ether (2f)

Was prepared from carvacrol using the method of Coolen et al. (1995). Clear oil (75%). IR (film, cm^−1^) 2958 (C-H), 1253 (CO-C). ^1^H NMR (400 MHz, CDCl_3_) δ 7.01 (d, 1H, J= 7.5 Hz, Ar-H), 6.70 (d, 1H, J= 7.5 Hz, Ar-H), 6.67 (s, 1H, Ar-H), 3.76 (s, 3H, OCH_3_), 2.84 (sept, 1H, J= 6.8 Hz, CH(CH_3_)_2_), 2.17 (s, 3H, Ar-CH_3_), 1.23 (d, 6H, J= 6.9 Hz, CH(CH_3_)_2_). ^13^C NMR (100 MHz, CDCl_3_): δ 157.6, 147.8, 130.4, 123.8, 117.9, 108.3, 55.0, 34.2, 24.1, 15.8. MS (EI) m/z [M]^+^ 164.

### Carvacryl ethyl ether (2g)

Was prepared from carvacrol using the method of Coolen et al. (1995). Clear oil (61%). IR (film, cm^−1^) 2960 (C-H), 1253 (CO-C). ^1^H NMR (400 MHz, CDCl_3_) δ 7.02 (d, 1H, J= 7.5 Hz, Ar-H), 6.70 (d, 1H, J= 7.5 Hz, Ar-H), 6.67 (s, 1H, Ar-H), 4.00 (q, 2H, J= 6.9 Hz, OCH_2_CH_3_), 2.83 (sept, 1H, J= 6.8 Hz CH(CH_3_)_2_), 2.18 (s, 3H, Ar-CH_3_), 1.39 (t, 3H, J= 6.9 Hz, OCH_2_CH_3_), 1.23 (d, 6H, J= 6.9 Hz, CH(CH_3_)_2_). ^13^C NMR (100 MHz, CDCl_3_): δ 157.0, 147.7, 130.4, 124.1, 117.8, 109.5, 63.3, 34.2, 24.1, 15.8, 15.0. MS (EI) m/z [M]^+^ 178.

### Thymyl methyl ether (3f)

Was prepared from thymol using the method of Coolen et al. (1995). Clear oil (68%). IR (film, cm^−1^) 2960 (C-H), 1257 (CO-C). ^1^H NMR (400 MHz, CDCl_3_) δ 7.08 (d, 1H, J= 7.6 Hz, Ar-H), 6.74 (d, 1H, J= 7.6 Hz, Ar-H), 6.66 (s, 1H, Ar-H), 3.80 (s, 3H, OCH_3_), 3.27 (sept, 1H, J= 6.9 Hz, CH(CH_3_)_2_), 2.32 (s, 3H, Ar-CH_3_), 1.19 (d, 6H, J= 6.9 Hz, CH(CH_3_)_2_). ^13^C NMR (100 MHz, CDCl_3_): δ 156.6, 136.2, 134.0, 125.7, 121.0, 111.3, 55.3, 26.4, 22.8, 21.3. MS (EI) m/z [M]^+^ 164.

### Thymyl ethyl ether (3g)

Was prepared from thymol using the method of Coolen et al. (1995). Clear oil (91%). IR (film, cm^−1^) 2958 (C-H), 1257 (CO-C). ^1^H NMR (400 MHz, CDCl_3_) δ 7.07 (d, 1H, J= 7.6 Hz, Ar-H), 6.72 (d, 1H, J= 7.6 Hz, Ar-H), 6.64 (s, 1H, Ar-H), 4.00 (q, 2H, J= 7.0 Hz, OCH_2_CH_3_), 3.28 (sept, 1H, J= 6.9 Hz, CH (CH_3_)_2_), 2.30 (s, 3H, Ar-CH_3_), 1.40 (t, 3H, J= 6.9 Hz, OCH_2_CH_3_), 1.19 (d, 6H, J= 6.9 Hz, CH(CH_3_)_2_). ^13^C NMR (100 MHz, CDCl_3_): δ 156.0, 136.1, 134.1, 125.8, 120.9, 112.3, 63.4, 26.6, 22.7, 21.3, 15.0. MS (EI) m/z [M]^+^ 178.

### 2-Hydroxy-3-methyl-6-(1-methylethyl) benzaldehyde (2h)

Was prepared from carvacrol using the method of Knight et al. (2005). Yellowish oil (31%). IR (film, cm^−1^) 3413 (OH), 2964 (C-H), 1666 (C=O). ^1^H NMR (400 MHz, CDCl_3_) δ 12.44 (s, 1H, Ar-OH, D_2_O exch.), 10.38 (s, 1H, COH), 7.29 (d, 1H, J= 7.7 Hz, Ar-H), 6.75 (d, 1H, J= 7.7 Hz, Ar-H), 3.59 (sept, 1H, J= 6.8 Hz, CH(CH_3_)_2_), 2.20 (s, 3H, Ar-CH_3_), 1.30 (d, 6H, J= 6.8 Hz, CH (CH_3_)_2_). ^13^C NMR (100 MHz, CDCl_3_): δ 195.1, 161.7, 150.2, 138.2, 124.3, 116.3, 115.5, 27.2, 24.2, 14.9. MS (EI) m/z [M]^+^ 178.

### 2-hydroxy-6-methyl-3-(1-methylethyl) benzaldehyde (3h)

Was prepared from thymol using the method of Knight et al. (2005). Yellowish oil (33%). IR (film, cm^−1^) 2966 (C-H), 1633 (C=O). ^1^H NMR (400 MHz, CDCl_3_) δ 12.29 (s, 1H, Ar-OH), 10.27 (s, 1H, COH), 7.29 (d, 1H, J= 7.6 Hz, Ar-H), 6.65 (d, 1H, J= 7.4 Hz, Ar-H), 3.31 (sept, 1H, J= 6.9 Hz CH(CH_3_)_2_), 2.54 (s, 3H, Ar-CH_3_), 1.21 (d, 6H, J= 6.9 Hz, CH(CH_3_)_2_). ^13^C NMR (100 MHz, CDCl_3_): δ 195.7, 160.9, 139.3, 134.0, 135.3, 121.4, 118.1, 26.1, 22.2, 17.8. MS (EI) m/z [M]^+^ 178.

### Carvacrylglycolic acid (2i),

Was prepared from carvacrol using the method of Riedl et al. (1998). White cotton like crystals (77%), mp 159–161 °C. IR (KBr, cm^−1^) 3413 (COOH), 1739 (COOH). ^1^H NMR (400 MHz, CD_3_OD) δ 7.01 (d, 1H, J= 7.6 Hz, Ar-H), 6.72 (d, 1H, J= 7.6 Hz, Ar-H), 6.64 (s, 1H, Ar-H), 4.62 (s, 2H, OCH_2_COOH), 2.81 (sept, 1H, J= 6.8 Hz, CH(CH_3_)_2_), 2.20 (s, 3H, Ar-CH_3_), 1.20 (d, 6H, J= 6.8 Hz, CH(CH_3_)_2_). ^13^C NMR (100 MHz, CD_3_OD): δ 173.0, 157.4, 148.9, 131.6, 125.3, 119.9, 110.7, 66.1, 35.2, 24.4, 15.9. MS (ESI) m/z [M–H]^−^ 207.

### Thymoxyacetic acid (3i),

Was prepared from thymol using the method of Riedl et al. (1998). White cotton like crystals (48%), mp 133–136 °C. IR (KBr, cm^−1^) 3429 (COOH), 1737 (COOH). ^1^H NMR (400 MHz, CD_3_OD) δ 7.06 (d, 1H, J= 7.7 Hz, Ar-H), 6.73 (d, 1H, J= 7.7 Hz, Ar-H), 6.60 (s, 1H, Ar-H), 4.61 (s, 2H, CH_2_COOH), 3.34 (sept, 1H, J= 6.9 Hz, CH(CH_3_)_2_), 2.26 (s, 3H, Ar-CH_3_), 1.19 (d, 6H, J= 6.9 Hz, CH(CH_3_)_2_). ^13^C NMR (100 MHz, CD_3_OD): δ 172.9, 156.4, 137.3, 135.3, 127.0, 123.0, 113.4, 66.2, 27.6, 23.2, 21.3. MS (ESI) m/z [M–H]− 207.

### Larvicidal activity

Two sets of carvacrol and thymol derivatives listed in Figs. 4–7 were tested for their in vivo larvicidal activities against *Ae. aegypti*, exhibiting a wide-ranging LC_50_. The rates of mortality were directly proportional to concentration. Their assessed lethal concentration for 50% mortality (LC_50_), as well as 95% confidence intervals (CI) expressed as ppm, are exhibited in Tables 1 and 2. Carvacrol and thymol exhibited LC_50_ of 51ppm (48 to 55) and 58ppm (54 to 63), respectively. Carvacrol derivatives exhibited LC_50_ ranging from 52 to 169ppm for field collected and from 39 to 113ppm for Rockefeller larvae, while thymol derivatives exhibited LC_50_ ranging from 34 to 465ppm for field collected and 18 to 101ppm for Rockefeller larvae.

Thymyl benzoate exhibited the highest overall larvicidal potency, with LC_50_ of 18ppm (14 to 25) for Rockefeller larvae, while thymoxyacetic acid exhibited the lowest larvicidal potency, with LC_50_ of 465ppm (426 to 513) for field-collected larvae.

Field collected strain exhibited certain resistance to most of the synthesized compounds than Rockefeller strain, related to the exposure of field mosquitoes to some of these compounds in aromatic plants.

### Computational methods

**Fig. 1.**
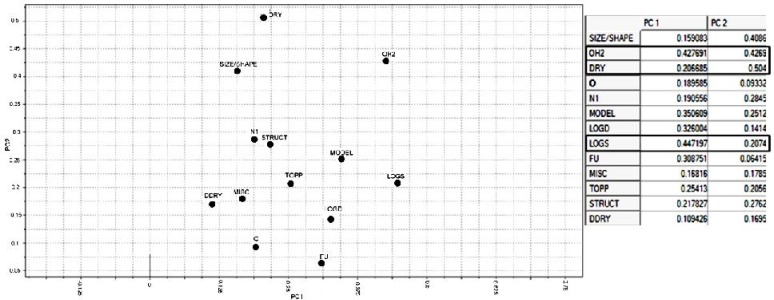
Blocks of descriptors

Blocks of descriptors exhibiting higher weights are shown in the Fig. 1, constructed taking into account the orthogonal properties of the CPCA: LogS (ADME descriptors), DRY and H2O.

**Fig. 2.**
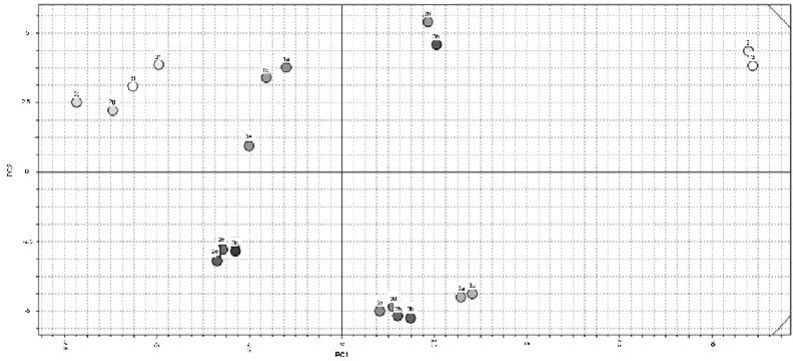
Positions of objects in relation to PC1 and PC2 components. Dark grey balls represent more active compounds, light grey balls represent less active compounds

Regarding CPCA methodology, 128 independent variables were used, and no biological data were considered as input to the model. The orthogonal properties of the CPCA algorithm were explored. The use of CPCA in decentralized process monitoring and diagnosis is originated from standard PCA scores and residuals. Two significant principal components (PCs) were obtained using a cross-validation methodology, which explains more than 68% of the total variance in larvicidal activity (Fig. 2).

**Fig. 3.**
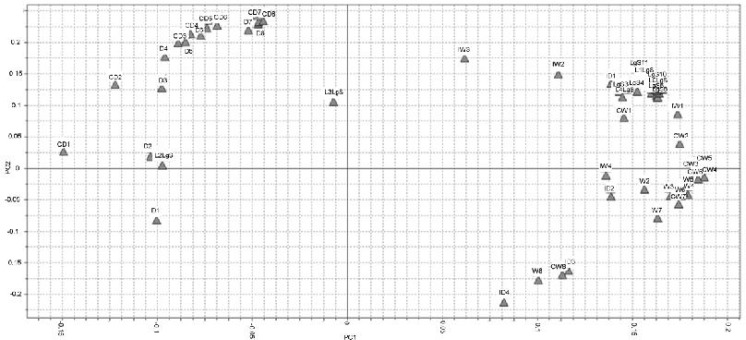
Loadings plot from PCA

PCA results were obtained using the H2O, DRY and LogS probes. Fifty-four descriptors were calculated. Once again, for both activities, approximately 73% of the total variance was explained by the PC1 and PC2 components. Fig. 3 represents the loading plot from PCA.

## Discussion

Carvacrol and thymol have previously exhibited moderate larvicidal activity. In view of this fact, our goals were to synthesize and evaluate the larvicidal activity of a diverse set of derivatives exhibiting a wide range of chemical physicochemical characteristics, which includes polarity, volume, hydrophobicity, and reactivity, to study the influence of such chemical characteristics in the larvicidal potency.

Derivatives 2a-i and 3a-i were synthesized by previously reported methods in reasonable yields and characterized by ^1^H and ^13^C NMR, FTIR spectroscopy, mass spectrometry and melting point. Replacement of the phenolic proton resulting in ether, ester and acetic acid derivatives were synthesized with the goal to examine the effects of such groups in modulating the larvicidal potency. Additionally, aldehyde groups at the α-position to the phenolic hydroxyl were synthesized.

Parent phenol monoterpenes (carvacrol and thymol) were used to synthesize their derivatives. Compounds 2a-i to 3a-i were synthesized by the routes depicted in Figs. 4–7. Carvacryl and thymyl esters were synthesized according to the literature with some modifications (Grodnitzky 2002, Dolly 2003, Ben Arfa 2006). Ester derivatives of carvacrol and thymol, i.e. carvacryl acetate, chloroacetate, trichloroacetate, and propionate, as well as, thymyl acetate, chloroacetate, trichloroacetate, propionate and benzoate were synthesized by reaction with acetic anhydride or acid halides in the presence of base (sodium acetate, triethylamine or NaH) in THF as solvent at room temperature.

Carvacrol and thymol exhibited similar potencies (51 and 58ppm for field collected, 47 and 46ppm for Rockefeller strains, respectively). Similarly, the acetate and the ethyl ether derivatives exhibited comparable potencies.

Substitution of the acidic proton of carvacrol by esters, ethers, and acetic acid resulted in either maintenance or reduction of potency. Ether derivatives 2f, 2g, 3f, and 3g exhibited up to 2.5-fold decrease in potency for field-collected strain, while no change in potency was observed for these compounds on Rockefeller larvae. Furthermore, carvacrylglycolic acid (2i) exhibited 2.5 to three orders of magnitude decrease in potency. The addition of a bulky benzoate group, yielding 2e, resulted in slight decrease in potency. However, the benzoate derivative was more potent than smaller groups, such as acetate or propionate. Similar results were previously achieved by substitution of the acidic proton of eugenol (Barbosa 2012).

Addition of chlorines to carvacryl acetate (LC_50_ = 93 and 72ppm for field collected and Rockefeller larvae, respectively), resulting on 2b and 2c led to a slight increase in potency (52 and 77ppm for field collected larvae and 39 and 54ppm for Rockefeller larvae, respectively).

Adding an aldehyde group at the ortho position of thymol phenolic hydroxyl resulted in about 1.5 orders of magnitude increase in potency. However, the presence of the aldehyde group at the ortho position of carvacrol resulted in minor potency decrease. Similar results were found in a previous work by our laboratory (Santos 2010). The addition of an aldehyde group at the ortho position of phenol resulted in about 1.5-fold increase in potency, while the same group added to the para position of catechol led to minor decrease in potency.

In the Consensus PCA, the analysis was performed by comparison of several descriptor blocks for the same object. In this study, thirteen groups of descriptors were calculated and plotted considering the two main components (PC1 and PC2), based on their weights. As observed in Fig 1, the blocks of descriptors: LogS, DRY and OH2 have the highest weights considering the two main PCs. The CPCA algorithm is basically comparable to the regular PCA. The blocks with high weight from CPCA, were selected and applied in PCA. Thus, 54 descriptors were used in the calculations of PC Analysis.

The PCA evaluations measured the 3D interaction energies computed using the LogS, DRY and H2O probes of GRID force field. The PCA method also aided to refine the data. The PC1 and PC2 components captured approximately 73% of the total variance from the original data.

In the scores plot (Fig. 2) the least active compounds are found in the higher left side of the graph, while the most active compounds are mostly found in the right side of the graph. Additionally, with the exception of 2e and 3e each derivative is found close to its constitutional isomer. Therefore, the contributions of the substituents for the larvicidal activity are similar for thymol and carvacrol derivatives.

The loadings plot (Fig. 3) highlighted the variables:

CW4-CW6–represent the hydrophilic surface divided by the total molecular surface. In other words, it is the hydrophilic surface per surface unit. Capacity factors are computed at eight dissimilar levels, the same energy levels used to calculate the hydrophilic volumes.
D7, D8–a probe called DRY is used by the software GRID to create 3D lipophilic fields. Similar to hydrophilic regions, hydrophobic regions are defined as the molecular envelope producing attraction by hydrophobic interactions. D1 to D8 are hydrophobic descriptors representing eight energy levels able to be calculated by Volfsurf. These levels can be associated to eight-energy range of hydrophobic interactions (From 0.2 to 1.6 kcal/mol).
CD1, CD2, CD8–is the quotient of the hydrophobic surface divided by the total molecular surface. Therefore, it is the hydrophobic surface per total surface. Capacity factors are computed at eight different levels, the same energy levels used to calculate hydrophobic volumes.
ID4–It is a measure of the unbalance between the center of mass of a compound and the centroid of its hydrophobic regions.

The Volsurf descriptors allows to represent by graphic the molecular interaction regions of interest, which is a substantial benefit in pharmacological studies as the descriptors are independent of the molecular alignment within the grid. The major advantage this method is the large amount of probes that are available. In addition, several functional groups may be represented, such as, hydrogen acceptor and donator probes (eg, water), constrained probes (eg, carbonyl probe), as well as hydrophobic probes.

Thymol derivatives were, to a certain extent, more efficient larvicides against *Ae. aegypti* than carvacrol derivatives, particularly to Rockefeller larvae. A withdrawing group (chlorine) added to thymyl acetate, resulting in thymyl chloroacetate (3b) led to an overall increase in potency. However, increasing the number of chlorines in the acetate group, resulting in thymyl tricholoroacetate (3c) did not increase potency.

The chemometric tools (CPCA and PCA) applied to carvacrol and thymol derivatives exhibited reasonable results. The variables outlined in this study indicate the presence of hydrophobic and hydrophilic regions in the molecular surface (mixed profile). However, hydrophobic interactions are more important and contribute to an increase the larvicidal activity. In a recent study published by our group (Scotti 2014) we performed chemometric studies (CPCA, PCA, and PLS) on a set of fifty-five larvicidal compounds against *Ae. aegypti*, including beyond the terpenes, the diterpenes, phenylpropanoids and phenol derivatives. The blocks of descriptors SIZE/SHAPE, DRY, and H2O exhibited higher weights in the CPCA analysis. In the present study, similar results were found, and the main blocks were LogS, DRY and H2O, demonstrating that for this series of compounds (thymol and carvacrol derivatives), aspects related to the hydrophobicity (LogS) are more important than the steric properties (SIZE/SHAPE) in explaining the larvicidal activity. In the same study the scores plot from PCA (generated with 48 descriptors, selected of the three main blocks) resulted in two principal components (PC1 and PC2) explaining over 62% of the total variance, whereas the present study (constructed with 54 descriptors, selected of the three main blocks) permitted to obtain a better model, in which the PC1 and PC2 explained approximately 73% of the total variance.

Evaluated compounds were active against third-instar larvae of *Ae. aegypti*. Field collected *Ae. aegypti* larvae exhibited more resistance to evaluated compounds than Rockefeller population.

## Conclusion

Substitution of the free hydroxyl function proved to modulate the larvicidal activity. The addition of an aldehyde group at the *ortho* position relative to the phenolic hydroxyl resulted in slight increase in larvicidal potency. Hydrophobicity plays an important role in the larvicidal activity of evaluated compounds. The addition of lipophilic groups to new designed compounds should prove to increase the larvicidal potency. Such studies are currently being conducted by our research group. In accordance with the present conclusions, we hope that these chemometrics and SAR data will be beneficial in the field of pest control and for the design of novel bioactive molecules.
